# The consumption of seaweed as a protective factor in the etiology of breast cancer: proof of principle

**DOI:** 10.1007/s10811-012-9931-0

**Published:** 2012-11-10

**Authors:** Jane Teas, Sylvia Vena, D. Lindsie Cone, Mohammad Irhimeh

**Affiliations:** 1 South Carolina Cancer Center, University of South Carolina, 915 Greene Street, 2nd Floor, Columbia, SC 29208 USA; 2 School of Medicine, South Carolina Cancer Center, University of South Carolina, 915 Greene Street, 2nd Floor, Columbia, SC 29208 USA; 3 Save Sight Institute, Sydney Hospital and Sydney Eye Hospital, Central Clinical School, University of Sydney, Sydney, NSW Australia

**Keywords:** Breast cancer, Clinical trial, CM10 ProteinChip, Surface-enhanced laser desorption/ionization–time-of-flight mass spectrometry (SELDI-TOF-MS), *Undaria pinnatifida*, Human urokinase-type plasminogen activator receptor (uPAR)

## Abstract

Daily consumption of seaweed has been proposed as a factor in explaining lower postmenopausal breast cancer (BC) incidence and mortality rates in Japan. This clinical trial assessed the impact of introducing seaweed- to non-seaweed-consuming American postmenopausal women. Fifteen healthy postmenopausal women were recruited for a 3-month single-blinded placebo controlled clinical trial; five had no history of BC (controls) and ten were BC survivors. Participants ingested ten capsules daily (5 g day^−1^) of placebo for 4 weeks, seaweed (*Undaria*) for 4 weeks, then placebo for another 4 weeks. Blood and urine samples were collected after each treatment period. Urinary human urokinase-type plasminogen activator receptor concentrations (uPAR) were analyzed by ELISA, and urine and serum were analyzed for protein expression using surface-enhanced laser desorption/ionization–time-of-flight mass spectrometry (SELDI-TOF-MS). Urinary creatinine standardized uPAR (in pg mL μg^−1^ creatinine) changed significantly between groups, decreasing by about half following seaweed supplementation (placebo 1, 1.5 (95 % CI, 0.9–2.1) and seaweed, 0.9 (95 % CI, 0.6–1.1) while placebo 2 returned to pre-seaweed concentration (1.7 (95 % CI, 1.2-2.2); *p* = 0.01, ANOVA). One SELDI-TOF-MS-identified urinary protein (*m*/*z* 9,776) showed a similar reversible decrease with seaweed and is reported to be associated with cell attachment. One serum protein (*m*/*z* 8,928) reversibly increased with seaweed and may be the immunostimulatory complement activation C3a des-arginine. uPAR is higher among postmenopausal women generally, and for BC patients, it is associated with unfavorable BC prognosis. By lowering uPAR, dietary seaweed may help explain lower BC incidence and mortality among postmenopausal women in Japan.

## Introduction

The relationship between the relative breast cancer (BC) risk and seaweed intake among humans is only now unfolding. A small body of research, both in vivo and in vitro, suggests seaweed may be useful in BC prevention (Funahashi et al. 1999; Teas et al. 1984; Yamamoto et al. 1987). Seaweeds are specifically used to treat tumors in Traditional Chinese Medicine and Japanese folk medicine. On a population level, those people for whom seaweed is a regular part of their diet, most notably in Japan, have dramatically lower rates of BC (Hebert and Rosen 1996; Kodama et al. 1991).

Epidemiologic studies done in Japan in the 1980s, before Westernized diets were common, reported that Japanese women had one out of three the rate of premenopausal BC and one out of nine the rate of postmenopausal BC (Reddy et al. 1980). Even today, BC incidence rates for women in Japan are 20/100,000 compared with the US average of 118/100,000 (US Cancer Statistics Working Group 2007). Although genetic predisposition has been proposed, when rates among migrants from Japan to the USA are compared, BC incidence almost doubles after 10 years of residence in the USA (20/100,000 to 35/100,000) (Shimizu et al. 1991) and continues to increase with each successive generation (LeMarchand et al. 1985). Japanese–American women who develop BC have significantly better survival rates than other American ethnic groups (Kanemori and Prygrocki 2005). On the other hand, Asian–American women over 50 years of age living in Los Angeles, especially Japanese–American women, have one of the most rapidly increasing BC incidence rates (Deapen et al. 2002). These data support the hypothesis that lifestyle changes and possibly gene–nutrient interactions are important in BC susceptibility.

Seaweed is a typical part of East-Asian diets, although consumption varies widely among individuals (Fukuda et al. 2007). Brown seaweeds have no land equivalents in terms of their specific components of fiber (alginate), primary carotenoid (fucoxanthin), sulfated polysaccharide (fucoidan and laminarin), and polyphenol defense compounds, each of which has been reported to have strong anti-cancer activity (Kang et al. 2012; Kotake-Nara et al. 2005; Koyanagi et al. 2003; Miao et al. 1999; Son et al. 2003).

Many in vivo and in vitro studies of dietary seaweed report decreased angiogenesis and increased apoptosis of tumor cells (Konishi et al. 2006; Koyanagi et al. 2003; Sekiya et al. 2005), inhibition of tumor cell adhesion and metastasis (Liu et al. 2005), and enhanced immune responses (Maruyama et al. 2006). Although Nishino et al. (2000) have investigated seaweed activation of the urokinase plasminogen system pathway, urokinase-type plasminogen activator receptor (uPAR) was not specifically studied. Based on the wide range of antitumor effects, we investigated the possibility that seaweed could affect uPAR concentrations in women who consume seaweed. The uPAR (CD87), is the membrane receptor for serine protease and is central to plasminogen activation and pericellular proteolysis. uPAR is an extracellular receptor involved in multiple signaling pathways important in maintaining homeostasis, directly interacting with 42 proteins, affecting the extracellular matrix (ECM), cell adhesion, inflammation, immune function (complement) activation, blood coagulation, and tissue repair (Eden et al. 2011). Increased concentrations have been shown to be associated with the presence of cancer, and increased cell surface shedding of uPAR is characteristic of more rapid cancer progression (Boonstra et al. 2011). We therefore included evaluation of uPAR as a possible biomarker for seaweed activity that might be related to BC prevention.

To further assess whether a dietary seaweed intervention could alter protein expression in urine and serum in a non-seaweed-consuming population of healthy postmenopausal women, we used surface-enhanced laser desorption/ionization–time of flight coupled with mass spectrometry (SELDI-TOF-MS). Proteomic analyses have been used to identify cancer biomarkers with high sensitivity and specificity, including those related to BC (Gast et al. 2009; van Winden et al. 2009). SELDI has also been shown to be sensitive enough to be used to identify changes in serum associated with the addition of a novel food (green tea) (Tsuneki et al. 2004).

## Materials and methods

### Study population

The Human Subjects Research Review Board of the US Army Medical Research and Materiel Command, the Palmetto Hospital Health Alliance (Columbia, SC), and the University of South Carolina Institutional Review Boards approved the study (IRB number, 99-104). The clinical study registration number is NCT01663792. Consent forms were reviewed verbally, and all participants gave written informed consent.

Inclusion criteria were: being healthy, postmenopausal (verified by follicle stimulating hormone (FSH; 23.0–116 mIU mL^−1^)), no allergies to seaweed, soy, shellfish or iodine; no current use of tobacco; no hormone replacement therapy or, for BC survivors, no chemotherapy or radiation treatments within the preceding 6 months and no history of cancer (other than BC or squamous cell skin cancer) within the previous 20 years; no current gastrointestinal disorders or diabetes; and omnivorous eating habits (including meat and dairy products more than twice per week), limit alcoholic intake to ≤1 drink (12 g alcohol)/week, and no oral antibiotics taken in the previous 3 months. Women agreed to eat their normal diet, avoiding seaweeds and to continue their habitual use of medications, vitamins, and supplements during the study.

Women were recruited through the Palmetto Health Tumor Registry, the South Carolina Comprehensive Breast Center registry, and the Psychosocial Oncology patient database of women who had indicated their interest in participation in BC research projects and had signed consent forms giving permission to be contacted. Once contacted, if eligible for study, the woman was mailed an Informed Consent form along with a confirmation letter of her scheduled appointment. Three women dropped out of the study after the initial contact but before commencing the study, leaving 15 healthy postmenopausal women. All 15 were mammographically negative for BC; five had never been diagnosed for BC (Controls), five had been treated for early (stages 0, I, or II) estrogen receptor-positive BC (ER+), and five had been treated for early estrogen receptor negative BC (ER−). Diagnosis of estrogen receptor status was confirmed by the treating physician.

### Study design

This single-blinded placebo-controlled study incorporated a 1-month placebo run in period (P1) followed by a 1-month treatment with seaweed (S) followed by a 1-month placebo washout period (P2). Identical capsules filled with maltodextrin were used as placebo. All participants ingested 5 g day^−1^ of the allocated treatment in ten 500-mg capsules.

### Adherence to protocol

At each clinic visit, women were asked about any significant health changes, including new medications or alcoholic beverages consumed. Urinary iodine was used to confirm adherence to the protocol during seaweed supplement period.

### Seaweed and placebo

*Undaria pinnatifida* (Harvey) Suringar (Japanese wakame) is one of the most popular dietary seaweeds eaten in Japan and Korea (Maruyama et al. 2003). The *U. pinnatifida* was harvested by Soriano from the Bahia Bustamante cove on the Patagonian coast of Argentina. We only used the sporophylls (Japanese mekabu), known to contain the highest amount of fucoidan (8 %; Soriano S.A., personal communication). Fucoidan is one of the water soluble components of seaweed that has shown high antitumor effects studies (Cumashi et al. 2007).

A 5-g serving of *Undaria* contained 21 kJ, 0.5 g protein, 0.4 g fat, 0.06 g carbohydrate, 2.55 g fiber, 269.6 mg potassium, and 153.2 mg sodium (Soriano 2008). Maltodextrin (Corn Products, Westchester, IL) was used as placebo, and 5 g/day provided 84 kJ.

### Encapsulation

*Undaria* was tested for *Escherichia coli*, *Salmonella*, *Staphylococcus aureus*, *Pseudomonas aeruginosa*, all of which were non-detectable, yeast and mold, aerobic recount, and moisture, met all safety standards (Proanalsis S.A. and Food Control S.A. Argentina). The seaweed was milled to a 300-μm powder (done on site at Gaiman by Soriano S.A., Argentina). Both the maltodextrin and seaweed were encapsulated into identical brown gelatin capsules (Vicofer, Argentina).

### Samples collection

Blood samples were drawn from fasting participants between 0630 and 1030 by venipuncture using SST vacutainer tubes. The blood was allowed to clot at 4 °C for 30 min, and then centrifuged at 1,500 rcf for 10 min before being aliquoted into 1 mL tubes. First morning urine samples were collected on the day of each clinic visit and aliquoted into 1 mL tubes (200 μL each). All samples were stored at −80 °C until analyses.

### Laboratory analyses

The Iodine Research Laboratory, Department of Endocrinology, Diabetes and Nutrition, Boston University School of Medicine analyzed the capsules for iodine content.

Iodine in urine, empty capsules, and finished capsules was analyzed using the ceritic-arsenic redox reaction. Samples were analyzed according to standard determination of total iodine protocol as outlined previously (Benotti et al. 1965). This used the reduction–oxidation reaction between ceritic and arsenite catalyzed by iodine. The iodine concentration was proportional to its catalytic activity. First, iodine was precipitated with perchloric acid, and the samples were digested with chloric acid. They were then measured spectrometrically at 420 nm (Autoanalyzer, Technicon Instrument, Inc). Calculations were based on an iodine standard curve. The urine results were calculated as μg of iodine dL^−1^ g^−1^ of creatinine.

Serum samples were analyzed for FSH at the Palmetto Health Richland Hospital Core Laboratory in Columbia, SC using standard direct chemiluminescent technology.

Urinary uPAR was analyzed at the University of South Carolina Cancer Research Center using uPAR immunoassay kit according to the manufacturer directions (R&D Systems, Minneapolis, USA, cat No. DUP00). The optical density was determined using a microtiter plate reader set at 450 nm (Packard SpectraCount, USA).

### SELDI-TOF-MS analysis: serum sample preparation

Samples were thawed and then prepared by adding 180 μL of denaturing buffer (8 M urea, 2 % CHAPS, 50 mM Tris, pH 9) to 20 μL of serum then vortexed for 30 min at 4 °C and diluted in 100 mM ammonium acetate (pH 4, 1:10) binding buffer. Fifteen microliters of sample was added to each spot and incubated for 1 h followed by a series of washes with binding buffer and deionized H_2_O. Sinapinic acid (SPA) was applied and arrays were analyzed.

### Urine sample preparation

Samples were thawed and vortexed briefly and then centrifuged at 10,000 rcf for 5 min at 4 °C. 160 μL of each sample was mixed with 60 μL of denaturing buffer (8 M urea, 2 % CHAPS, 50 mM Tris, pH 9) in 1.5 mL tubes then vortex and incubated for 30 min at 4 °C. The pH was then set to 4.0 using NH_4_OH and H_3_PO_4_. SELDI CM10 (weak cation exchange) chip was then equilibrated with 100-μL binding buffer (100 mM NH_4_Oac) and shook for 5 min. Sample mix (200 μL) was then combined with 200-μL binding buffer and vortexed briefly. Binding buffer was then discarded from chips and 190-μL sample mix/binding buffer was added to two spots for replication. Chips were then covered with Parafilm and shaken for 1 h. The samples were then removed from chips, washed three times with 100-μL binding buffer and shaken for 5 min before they were washed twice with 100 μL ddH_2_O, shook for 2 min and air dried. That was followed by applying 1 μL energy absorbing material (SPA) then dried twice. The chips were finally light protected until analysis.

### SELDI-TOF-MS analysis

Samples were analyzed at the Molecular Epidemiology and Toxicology Laboratory at the University of California, Berkeley. CM10 ProteinChip arrays were purchased from Ciphergen Biosystems (Fremont) and prepared as described elsewhere (Hegedus et al. 2005) and then analyzed using SELDI-TOF-MS ProteinChip Reader (Series PBSII, Ciphergen Biosystems) with laser intensity setting of 200 (arbitrary units), and detector sensitivity at 8, collecting peak data at every five positions along the spot surface. Mass spectra were generated in the *m*/*z* range of 1,500–25,000. The *m*/*z* value for each of the peaks was determined using external calibration with known standards using [Arg8]-vasopressin (1,084.25 + 1H), somatostatin (1,637.9 + 1H), dynorphin A [209–225] (porcine; 2,147.5 + 1H), ACTH [1–24] (human; 2,933.5 + 1H), insulin B-chain (bovine; 3,495.94 + 1H), insulin (human recombinant; 5,807.65 + 1H), and hirudin (recombinant; 6,963.52 + 1H). The Ciphergen Express (Ciphergen Biosystems) software collected peak data, and each peak was analyzed according to the following parameters: first pass of 5, second pass of 2, 0.8 % peak width named same peak, and 25 % min^−1^ all spectra.

### Statistical analysis

Simple descriptive statistics were used to characterize the study sample. Our statistical models used all available data without imputation, allowing us to use the standard intention-to-treat approach (Rothman 1998). To test the main study hypotheses, a repeated measures analysis of variance (ANOVA) was conducted using repeated measures ANOVA (GraphPad Prism 5) with Tukey’s multiple comparison tests to determine differences between treatment groups. Statistical analysis clusters were generated by Ciphergen Protein Chip Software Version 3.1 (Bio-Rad) from 1.5 to 25 kDa with the following settings: first-pass signal-to-noise ratio (S/N) of 5, second-pass S/N of 2, minimum peak threshold of 25 %, and a cluster mass window of 0.8 %. Statistical analysis was performed using the same software. Only *m*/*z* values significantly different from the background are reported. All samples were analyzed in duplicates. Individuals in each group of subjects (control, ER+, or ER−) were paired for analysis by group then by treatment arm (P1, S, and P2). Mann–Whitney *U* test was applied to generate *p* values. Analysis of variance was used to compare health and demographic variables. A *p* value of 0.05 was considered significant.

## Results

Demographic variables for the study participants are presented (Table 1) and show no significant differences. The health variables in Table 2 show cancer survivors had higher screening FSH when compared with controls, indicating possible hormonal differences. It was also notable that the ER+ had lower BMI than the other two groups.

**Table 1:** Demographic variables for healthy postmenopausal women at baseline compared by disease status

	Control	ER+	ER−
*n*	5	5	5
Age years (95 % CI)	59.8 (56–64)	60.4 (55–66)	58 (50–66)
Ethnicity			
African American	2	0	4
European American	3	5	1
Education			
High school	1	0	1
Some college or Technical degree	1	2	1
Bachelors or higher	3	3	3
Family income (annual)			
$20–49,000	2	1	1
$50–99,000	2	1	3
>$100,000	1	2	0
Do not know	0	1	1

**Table 2:** Comparison of health variables for healthy postmenopausal women at baseline compared by disease status

	Controls	ER+	ER−
Screening FSH (mIU mL^−1^; 95 % CI)	57.4 (45.4, 69.4) ^a^	72.5 (34.4, 110.7) ^b^	78.3 (59.5, 97.1) ^b^
Systolic blood pressure (mmHg; 95 % CI)	134.6 (119.3, 149.9) ^a^	112.4 (97.6, 127.2) ^b^	127.2 (114.9, 139.5) ^a^
Diastolic blood pressure (mmHg; 95 % CI)	96.6 (87.1, 106.1) ^a^	74.2 (63.5, 84.9) ^b^	89.8 (82.0, 97.6) ^a^
BMI (95 % CI)	34 (27, 41) ^a^	24 (18, 30) ^b^	32 (23, 41) ^a^

*BMI* body mass index = weight (kg)/height (m)^2^

Different superscript letters within each column indicate significant differences between groups (*p* = 0.05; i.e., a vs. b is significant whereas a vs. a is not)

The iodine content in each 0.5 g capsule (*n* = 4) averaged 38.5 μg. Urinary iodine excretion standardized for creatinine (in μg dL^−1^ g^−1^ of creatinine) increased slightly during seaweed supplementation, from 0.04 (95 % CI, 0.02–0.06) after P1 to 0.05 (95 % CI, 0.03–0.06) on S returning to 0.03 (95 % CI, 0.02–0.04) on P2, indicating adherence to the protocol, but also that this low iodine-containing seaweed had little effect on iodine metabolism. These trends were the same for women taking thyroid hormone medication (*n* = 5) and those not taking thyroid medications (*n* = 10).

**Fig. 1:**
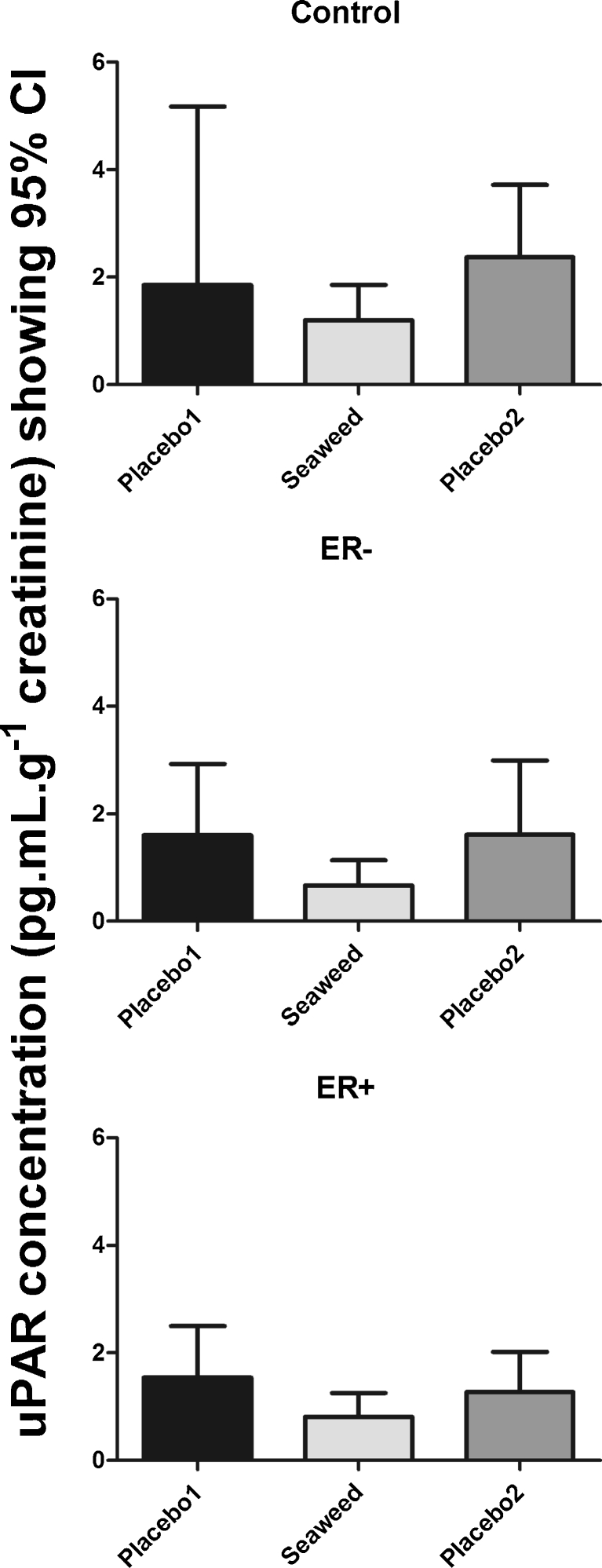
Urinary uPAR concentrations compared by BC history and treatment group (*error bars* indicate 95 % CI)

Urinary creatinine-standardized uPAR concentrations decreased by almost 50 % following seaweed supplementation that was reversed after the second placebo treatment. The uPAR concentrations (in pg mL μg^−1^ of creatinine) were P1, 1.5 (95 % CI, 0.9–2.1); S, 0.9 (95 % CI, 0.6–1.1); and P2, 1.7 (95 % CI, 1.2–2.2; *p* = 0.01, ANOVA). These trends were observed in each group, regardless of BC history (Fig. 1).

**Fig. 2:**
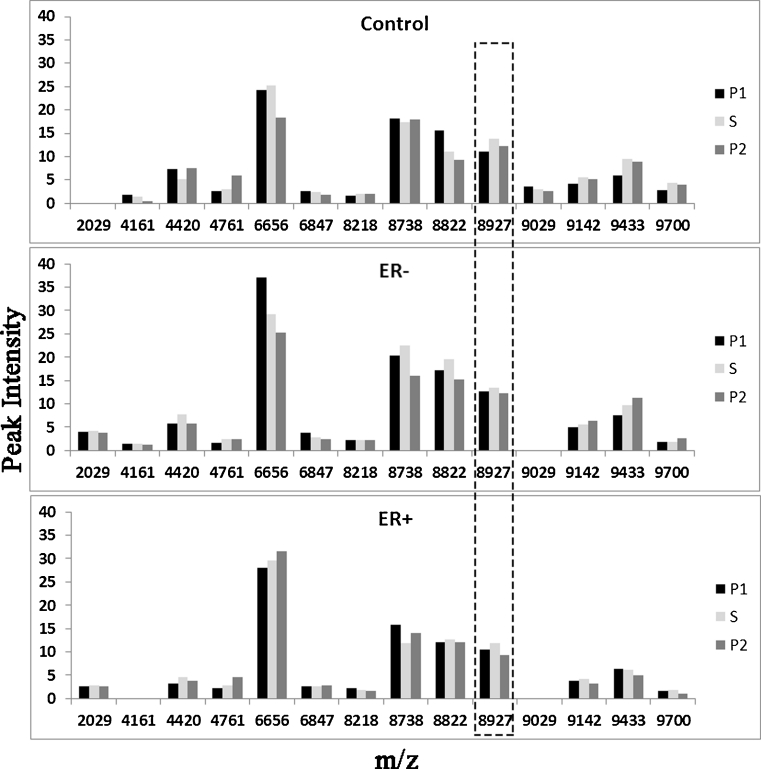
Comparison of differentially expressed serum proteins (*m*/*z*) and their relative intensities by BC history and treatment group

**Fig. 3:**
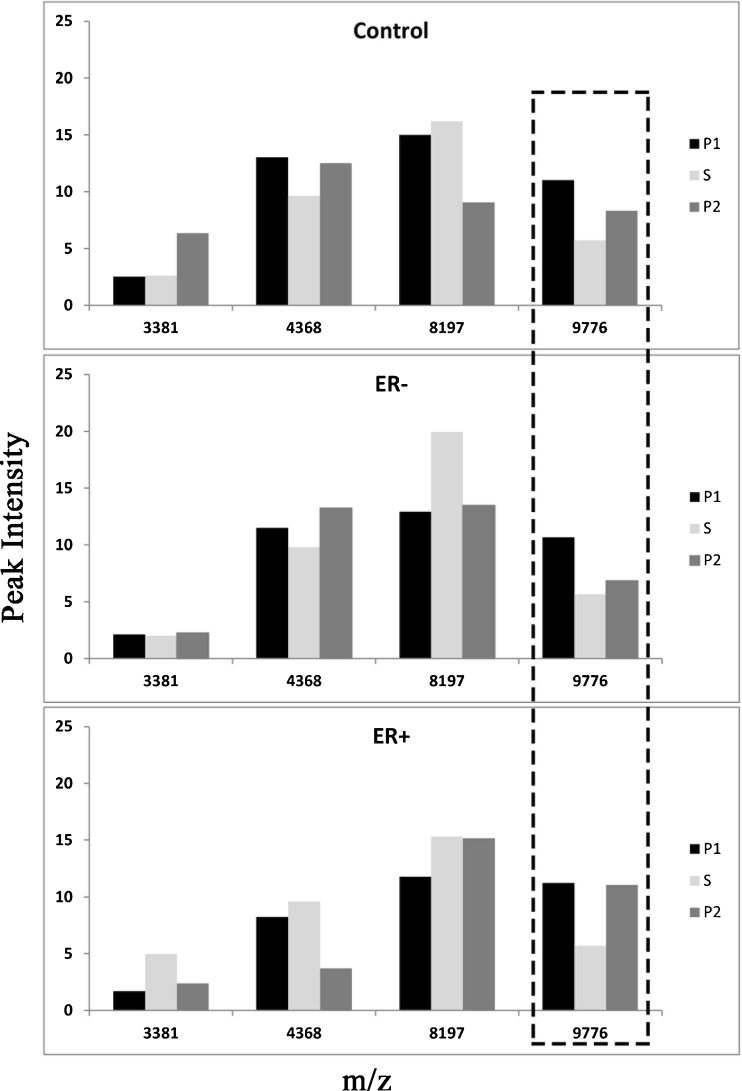
Comparison of differentially expressed urine proteins (*m*/*z*) and their relative intensities by BC history and treatment group

The SELDI-MS-TOF data identified a total of 14 proteins that were differentially expressed in serum (Fig. 2). Only one serum protein reversibly changed in all three groups with seaweed supplementation (*m*/*z* 8,927). Fewer urinary proteins were identified using SELDI (Fig. 3), and only one (*m*/*z* 9,776) showed a reversible response to seaweed, increasing slightly, but significantly.

## Discussion

We first proposed that seaweed intake could be a factor in BC prevention in 1981 (Teas 1981), and although the evidence from hundreds of research studies have supported seaweed as important in a host of biological pathways. Our study provides evidence that 5 g day^−1^ of brown seaweed (*Undaria*) stimulated significant changes in urinary uPAR and slight changes in two other proteins. For the relatively small amount of seaweed (5 g day^−1^) to result in a 50 % reduction in uPAR is remarkable. If supported in other studies, it gives a clear mechanism for explaining the various preventive and treatment-related effects of seaweed supplementation. At the very least, it gives a plausible mechanism for explaining the diverse findings of other studies, which include cell adhesion and detachment from the ECM of cancer cell leading to migration and metastasis (Hildenbrand and Schaaf 2009).

In many ways, it is difficult to imagine how a mere 5 g day^−1^ could have such a profound effect on systemic concentrations of uPAR and specific protein expression. One possibility is that seaweed, like other dietary fibers, is known to increase the mucus bilayer that protects the colonic mucosa, stimulating mucus secretion and thickness (Brownlee et al. 2005). Other suggested consequences of dietary seaweed fiber include increased stool bulk which would provide protection from dietary toxins and carcinogens and reduction in amylase activity leading to decreased glycemic response.

It is interesting that powdered seaweed (1 %, *w*/*v*) but not seaweed extracts were responsible for almost 100 % inhibition of amylase and pepsin digestive enzyme activity in vitro (Bobin-Dubigeon et al. 1997). Small water-soluble molecules which would be lost in the production of seaweed extracts were thought to be responsible for the inhibition of protease activities. Thus, the small amount of dietary seaweed used in our study and consumed in Japan might be expected to have a profound effect in humans.

Our study also provides a possible rationale for understanding why BC in non-seaweed-consuming Western populations increases with age, but remains more or less stable for Japanese women (Reddy et al. 1980). uPAR increases after menopause (Chung et al. 1998), and if seaweed, on a population level in Japan, has a significant impact on uPAR concentrations, it may be the basis for the stable rates.

Our investigation using SELDI was interesting. Several of proteins with high intensity have also been identified as important in BC (Gast et al. 2009). uPAR has an *m*/*z* protein size beyond the range we measured, but two proteins we identified changed with seaweed. Serum protein (*m*/*z* 8,927) has been identified as complement activation C3a des-arginine and may indicate stimulation of an immune response that has also been detected in subjects with early BC (Becker et al. 2004). In the seaweed literature, enhanced immune function has been reported in vitro (Kim and Joo 2008) and in vivo (Kandasamy et al. 2012; Leonard et al. 2012). Increased expression of *m*/*z* 8,928 may be associated with the observed enhanced immune function.

The urinary protein *m*/*z* 9,776 has not been identified as important in BC but it has been identified as a hormone-responsive protein important in decidualization of the endometrial stromal matrix preceding embryo implantation (Oh et al. 2008). This progesterone-responsive protein increased with seaweed. In a study of conception rates in heat-stressed cows, seaweed supplementation with a different brown seaweed (*Ascophyllum*) was associated with more than a 7-fold increase in successful pregnancies (Kellogg et al. 2006). This suggests that seaweed supplementation may influence cell surface signaling receptors for steroid hormones.

As BC in Japan is characterized by higher rates of the more favorable ER+ type, we anticipated that women with a history of ER− BC might have different protein responses to dietary seaweed. There were only minor differences in protein expression by BC history, and possibly the variations might reflect underlying differences in BC susceptibility. Further research should be conducted to define whether or not these minor differences reflect meaningful biological variations.

Seaweeds are consumed daily in Japan and are considered to be important in maintaining optimal health. The actual amount of seaweed consumed is somewhat problematic. Several different kinds of seaweed are eaten daily in a variety of forms and dishes and are consumed dried in sushi wrappers, fresh or rehydrated as a side dish, and used as flavor condiments as well as the basis for broth-based (dashi) flavoring added to other foods. It is therefore difficult to estimate the actual daily intake of seaweed. Our best guess is that 5 g/day may approximate the mean Japanese daily intake. A typical serving of the kind of seaweed used in this study, wakame (*Undaria*), has been estimated at 1 g by the Japanese Ministry of Health (Ministry of Health of Japan 2004). Thus our study could overestimate the contribution of *Undaria* to the average Japanese consumer, but as a pilot study, we propose that it combines the mean dose of seaweed and isolates a particular kind of seaweed, so the effects we report can be attributed to one seaweed species.

Our primary reason for using whole seaweed rather than a purified fraction is that people in Japan consume seaweed, and it is this dietary preference that appears to be associated with multiple health benefits (MacArtain et al. 2007). The combination of various bioactive components in whole seaweed may be greater than the sum of the individual parts, were they to be purified and tested individually. Many previous in vitro and in vivo studies have used hot or cold water extracts of *Undaria*, and have proposed that fucoidan, a water-soluble sulfated polysaccharide primarily found in brown seaweeds, as the most bioactive component (Cumashi et al. 2007). In previous studies we have demonstrated that *Undaria* fucoidan is absorbed by humans, despite its large molecular size (Irhimeh et al. 2005). Although it would be interesting to have used such an extract in our study, it would have required obtaining approval to use the extract as an Investigational New Drug, which would have taken extensive and costly pretesting evaluation to register with the US Food and Drug Administration. As an alternative, we chose to use high-fucoidan seaweed, using only the sporophyll of *Undaria*, collected at the peak of the summer harvest season. These sporophylls consistently contain 8 % fucoidan/dry weight and are also low in iodine (approximately 80 μg g^−1^), and 5 g day^−1^ would be well under the tolerable upper intake level of 1,100 μg/day (National Research Council 2001). In addition, no adverse effects have been recorded in our previous studies using this 5 g day^−1^ dose of *Undaria* sporophylls (Teas et al. 2009; Teas and Irhimeh 2012).

One of our study strengths was that it evaluated the introduction of seaweed to a non-seaweed-consuming population, and despite the women continuing with their normal diet, significant changes in protein expression were observed. The reversibility of the changes in the urinary concentration of uPAR and of the two SELDI-identified proteins strongly suggests the changes were attributable to the inclusion of seaweed. As any small proof of principle study, our small sample size was a limitation. However, finding significant differences bodes well for future studies.

Data from uPAR concentration changes indicate that seaweed affects ECM, which is known to influence cell surface signaling, cell adhesion, and growth factor communication and responsiveness in breast tissue. Additional data from SELDI proteomic analysis further supports the role of seaweed supplementation in modulating proteins known to affect cell adhesion, immune response, and inflammatory responses. These effects may be mediated through the ECM and be important in maintaining healthy structure and function in breast cells. In particular, lower uPAR concentrations could reduce the likelihood of breast tissue transformation and cancer cell proliferation and invasiveness.

The role of dietary seaweed in lowering uPAR may therefore be critical in explaining differences in postmenopausal BC incidence and mortality between Japanese women and women in other developed countries. The possibility that dietary seaweed could decrease uPAR is especially important in that there are no anti-uPAR drugs currently available (Degryse 2011). Finding that seaweed can have a significant effect on uPAR could be helpful both in designing future drugs and dietary interventions for people with cancer.
